# Supplementary material for: Aptamers with Self-Loading Drug Payload and pH-Controlled Drug Release for Targeted Chemotherapy
Source: Pharmaceutics. 2021 Aug 7;13(8):1221. doi: 10.3390/pharmaceutics13081221 (PMC8398837; doi:10.3390/pharmaceutics13081221)
Supplement: Supplementary file 1 [file pharmaceutics-13-01221-s001.zip › pharmaceutics-1326543-supplementary.pdf]

pharmaceutics

Supplementary Material: Aptamers with Self-Loading Drug Payload and pH-Controlled Drug Release for Targeted Chemotherapy

Zihua Zeng, Jianjun Qi, Quanyuan Wan and Youli Zu

Materials and Methods

General information of chemistry experiments

All reagents were purchased from Sigma-Aldrich (St. Louis, MO) and used without further purification. Column chromatography was performed on silica gel 60 (70-230 Mesh). Analytical thin-layer chromatography (TLC) was conducted on glass plates coated with silica gel 60 (F- 254). Proton (1H) and Carbon (13C) NMR spectra were obtained on a Bruker 300 MHz magnetic resonance spectrometer using TMS as internal standard. NMR chemical shifts (δ) are reported in ppm. Mass spectra (ESI-MS data) were collected on an LCQ Fleet™ Ion Trap Mass Spectrometer (Thermo, USA).

Experimental Section

Synthesis and characterization

Di-tert-butyl 3,3′-((2-amino-2-((3-(tert-butoxy)-3-oxopropoxy)methyl)propane-1,3-diyl)bis(oxy))dipropionate (compound 2)[1]. A flask containing a solution of tris base (1.21 g, 10.0 mmol) in DMSO (2 mL) was flushed by nitrogen, followed by addition of 5N NaOH (0.2 mL) with a syringe, and tert-butyl acrylate (5.0 mL, 34 mmol) was added dropwise at 0°C. The reaction solution was allowed to reach room temperature (RT) and then incubated overnight with stirring. The resulting compounds were extracted with EtOAc and washed with brine. The organic layer was dried with sodium sulfate and the solvent was removed under reduced pressure. The raw compound was further purified with a silica gel column, eluted with EtOAc/Hexane=1/1, and yielded 2.54 g of compound 2 as a colorless oil with a yield of 50.3%. TLC: Rf=0.4 (EtOAc). 1H NMR (CDCl3, 300 MHz): δ 1.45 (s, (CH3)3C, 27H); 2.46 (t, CH2CO, J=6.4 Hz, 6H); 3.32 (s, CCH2O, 6H); 3.65 (t, OCH2CH2, J=6.4 Hz, 6H) (Figure S2). 13C NMR (CDCl3, 75 MHz): δ 170.94, 80.43, 72.88, 67.13, 55.95, 36.33, 28.11 (Figure S3). ESI-MS: 506 (M+H)+ (Figure S1).

Di-tert-butyl 3,3'-((2-((((9H-fluoren-9-yl)methoxy)carbonyl)amino)-2-((3-(tert-butoxy)-3-oxopropoxy)methyl) propane-1,3-diyl)bis(oxy))dipropionate (compound 3) [2]. Compound 2 (505 mg, 1 mmol) was dissolved in a mixture solvent of 1,4-dioxane (5 mL) and 10% NaHCO3 aqueous solution (3 mL), followed addition of Fmoc-Cl (257 mg, 1 mmol). The reaction solution was stirred at RT overnight. Then, the reaction solution was extracted with ethyl acetate, washed with brine, and dried over sodium sulfate. The solvent was removed under reduced pressure. The residue was purified with a silica gel column, eluted with hexane/ethyl acetate from 4/1 to 2/1 to give 0.65 g of compound 3 with a yield of 89.4%. TLC: Rf=0.54, Hexane/EtOAc=2/1. 1H NMR (CDCl3, 300 MHz): δ1.44 (s, (CH3)3C, 27H); 2.45 (t, CH2CH2O, J= 6.2 Hz, 6H); 3.65 (m, OCH2CH2, 12H); 4.5-4.1 (m, 3H); 5.36 (bs, NH, 1H); 7.29 (td, 2H, J1=7.2 Hz, J2=1.2Hz), 7.63 (d, 2H, J=7.2Hz), 7.76 (d, 2H, J=7.2Hz) (Figure S5). 13C NMR (CDCl3, 75 MHz): δ 170.85, 144.13, 141.27, 127.60, 127.06, 125.21, 125.21, 119.90, 80.47, 69.33, 67.07, 66.36, 58.78, 47.24, 36.23, 28.10 (Figure S6). ESI-MS: 750 (M+Na)+ (Figure S4).

3,3′-((2-((((9H-fluoren-9-yl)methoxy)carbonyl)amino)-2-((2-carboxyethoxy)methyl)propane-1,3-diyl)bis(oxy))dipropionic acid (compound 4) [3]. To compound 3 (3.07g, 4.22 mmol), 32 mL of 95% TFA-5% H2O solvent was added and stirred at RT for 1 h. The solvent was removed under reduced pressure and produced compound 4 as a colorless oil. The residue was directly used in next step reaction without further purification. The sample to be

used for NMR analysis was purified using semi-preparative HPLC. TLC: Rf=0.54, hexane/ethyl acetate=2/1. 1H NMR (CD3OD, 300 MHz): δ2.48 (s, CH2CH2O, 6H); 3.65 (s, OCH2CH2, 12H); 4.5-4.1 (m, 3H); 7.45-7.25 (m, 4H), 7.65 (d, 2H, J=7.3Hz), 7.80 (d, 2H, J=7.3Hz) (Figure S8). 13C NMR (CD3OD, 75 MHz): δ 174.05, 144.07, 141.26, 127.37, 126.85, 124.76, 119.58, 68.72, 66.64, 65.94, 34.33 (Figure S9). ESI-MS: 582 (M+Na)+ (Figure S7).

Di-tert-butyl 9-((((9H-fluoren-9-yl)methoxy)carbonyl)amino)-9-((3-(2-(tert-butoxycarbonyl)hydrazineyl)-3-oxopropoxy)methyl)-4,14-dioxo-7,11-dioxa-2,3,15,16-tetraazaheptadecanedioate (compound 5). To the residue (compound 4) DMF (23 mL), DIEPA (5 mL) and HBTU (5.0 g, 13.2 mmol) were added and stirred at RT for 1 h. Then, tert-buty carbazate (2.0 g, 15.15 mmol) was added with continued stirring at RT overnight. After the reaction was completed, the solvent was removed under reduced pressure. The residue was extracted with ethyl acetate, washed with brine, and dried over sodium sulfate. After removing the solvent, the residue was purified with a silica gel column and eluted with ethyl acetate/methanol=10/1 to give 2.3 g of pure compound 5 with a yield of 60.5%. TLC: Rf=0.54, Hexane/Ethyl acetate=2/1. 1H NMR (CDCl3, 300 MHz): δ 1.45 (s, 27H), 2.48 (s, CH2CH2O, 6H); 3.72 (s, OCH2CH2, 12H); 4.5-4.1 (m, 3H); 5.41 (s, 1H), 6.86 (s, 3H), 7.30 (t, 2H), 7.41 (t, 2H), 7.60 (d, 2H, J=7.3Hz), 7.77 (d, 2H, J=7.3Hz), 8.67 (s, 3H) (Figure S11). 13C NMR (CDCl3, 75 MHz): δ171.20, 155.91, 141.34, 127.74, 127.15, 120.00, 81.82, 70.14, 66.98, 47.21, 34.54, 28.17 (Figure S12). ESI-MS: 924 (M+Na)+ (Figure S10).

Di-tert-butyl 9-amino-9-((3-(2-(tert-butoxycarbonyl)hydrazineyl)-3-oxopropoxy)methyl)-4,14-dioxo-7,11-dioxa-2,3,15,16-tetraazaheptadecanedioate (compound 6). Compound 5 (2.48 g, 2.75 mmol) was dissolved in DCM (16 mL) and 4 mL of piperidine and stirred at RT for 1 h. The solvent was removed under reduced pressure. The residue was purified with a silica gel column, eluted with ethyl acetate/methanol=10/1 to 5/2 to give 1.07 g of compound 6 as a yellow residue with a yield of 57.2%. TLC: Rf=0.57, ethyl acetate/methanol =5/1. 1H NMR (CDCl3, 300 MHz): δ 1.45 (s, 27H), 2.51 (t, CH2CH2O, 6H, J=5.4); 3.69 (s, OCH2CH2, 6H); 3.83 (t, 6H, J=5.4Hz), 6.56 (s, 3H), 8.24 (t, 3H), 9.40 (s, 2H) (Figure S14). 13C NMR (CDCl3, 75 MHz): δ 172.09, 156.32, 82.32, 69.33, 67.08, 60.24, 34.57, 28.09 (Figure S15). ESI-MS: 680 (M+H) +, 702 (M+Na) + (Figure S13).

3,3'-((2-amino-2-((3-(2-((Z)-1-(4-((4-amino-5-hydroxy-6-methyltetrahydro-2H-pyran-2-yl)oxy)-2,5,12-trihydroxy-7-methoxy-6,11-dioxo-1,2,3,4,6,11-hexahydrotetracen-2-yl)-2-hydroxyethylidene)hydrazineyl)-3-oxopropoxy)methyl)propane-1,3-diyl)bis(oxy))bis(N'-((Z)-1-(4-((4-amino-5-hydroxy-6-methyltetrahydro-2H-pyran-2-yl)oxy)-2,5,12-trihydroxy-7-methoxy-6,11-dioxo-1,2,3,4,6,11-hexahydrotetracen-2-yl)-2-hydroxyethylidene)propanehydrazide) (compound 1, TNM-DOX) [4]. Compound 6 (8 mg, 0.012 mmol) was dissolved in 1 mL of a mixture solvent of trifluoroacetic acid/methylene chloride (1/1) and stirred for 1 h at RT. Then, the solvent was removed under reduced pressure to produce oil in quantitative yield. The residue was used in next step reaction without further purification.

The above residue was added into a mixture solvent of methanol/acetic acid/pyridine (10/0.1/0.1, v/v) (2 mL). DOX (28 mg, 0.048 mmol) was added and the reaction solution was stirred overnight at RT. The reaction mixture was loaded directly onto a Sephadex LH-20 column and eluted with methanol. The first 5-part dark red band (0.5 mL each part) was collected and the solvent was removed using a speedVac concentrator to yield 3.5 mg of compound 1 with a yield of 15.2%. ESI-MS: 1955.7 (M+H) +, 978.8 (M+2H) 2+, 652.5 (M+3H) 3+ (Figure S16).

4.3. HPLC analysis

HPLC analysis was performed in a Varian 920-LC Liquid Chromatograph (Varian, USA) with a built-in auto-injector and UV-Vis detector. A Kinetex C18 column (100X4.6mm, 2.6μm, Phenomenex, USA) was used at a flow rate of 1 mL/min; Mobile phase A was 0.1% acetic acid in water; mobile phase B was acetonitrile; linear gradient was from 0 to 8 min, 0-85% of B. HPCL separation was monitored using the UV-Vis detector at 260 nm.

Reference:

1. Cardona, C. M.; Gawley, R. E. An improved synthesis of a trifurcated newkome-type monomer and orthogonally protected two-generation dendrons. *J. Org. Chem.* **2002,** *67*, 1411-1413.
2. Maegawa, T.; Fujiwara, Y.; Ikawa, T.; Hisashi, H.; Monguchi, Y.; Sajiki, H. Novel deprotection method of Fmoc group under neutral hydrogenation conditions. *Amino Acids* **2009,** *36*, 493-499.
3. Nagel, L.; Budke, C.; Dreyer, A.; Koop, T.; Sewald, N. Antifreeze glycopeptide diastereomers. *Beilstein J. Org. Chem.* **2012,** *8*, 1657-1667.
4. van der Poll, D. G.; Kieler-Ferguson, H. M.; Floyd, W. C.; Guillaudeu, S. J.; Jerger, K.; Szoka, F. C.; Frechet, J. M. Design, synthesis, and biological evaluation of a robust, biodegradable dendrimer. *Bioconjug. Chem.* **2010,** *21*, 764-773.

The mass spectrum, ^{1}H NMR, and ^{13}C NMR spectra of the chemical intermediates

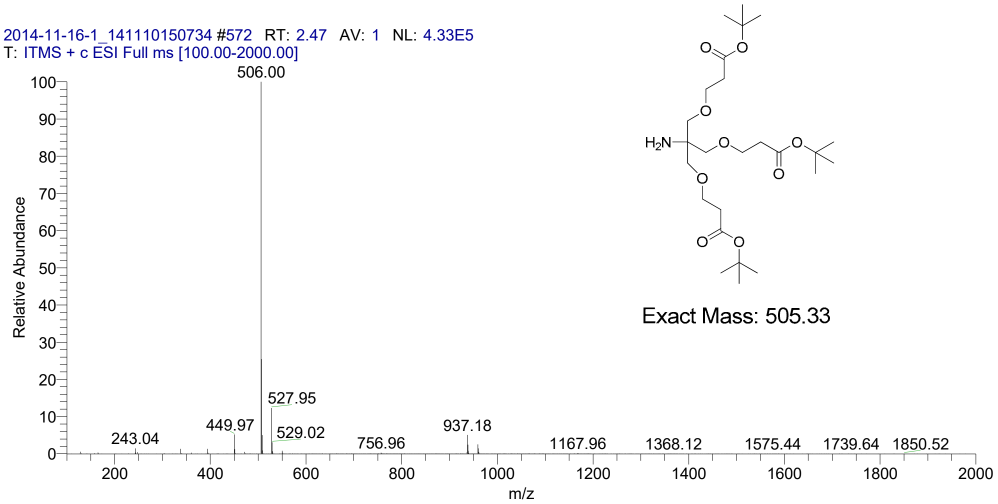

Figure S1. The mass spectrum of compound 2.

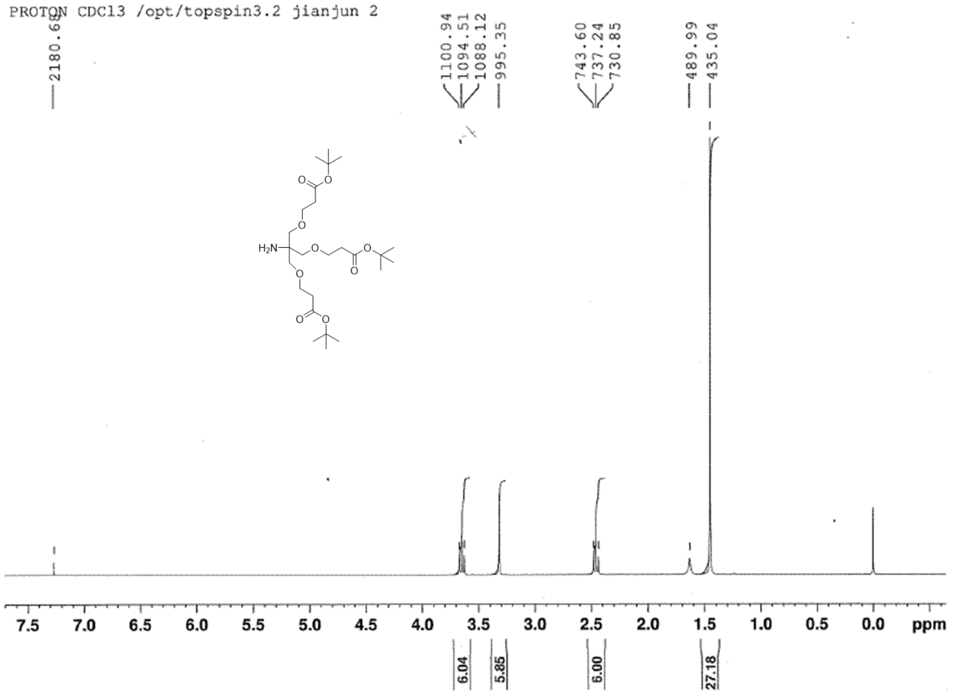

Figure S2. The 1HNMR spectrum of compound 2.

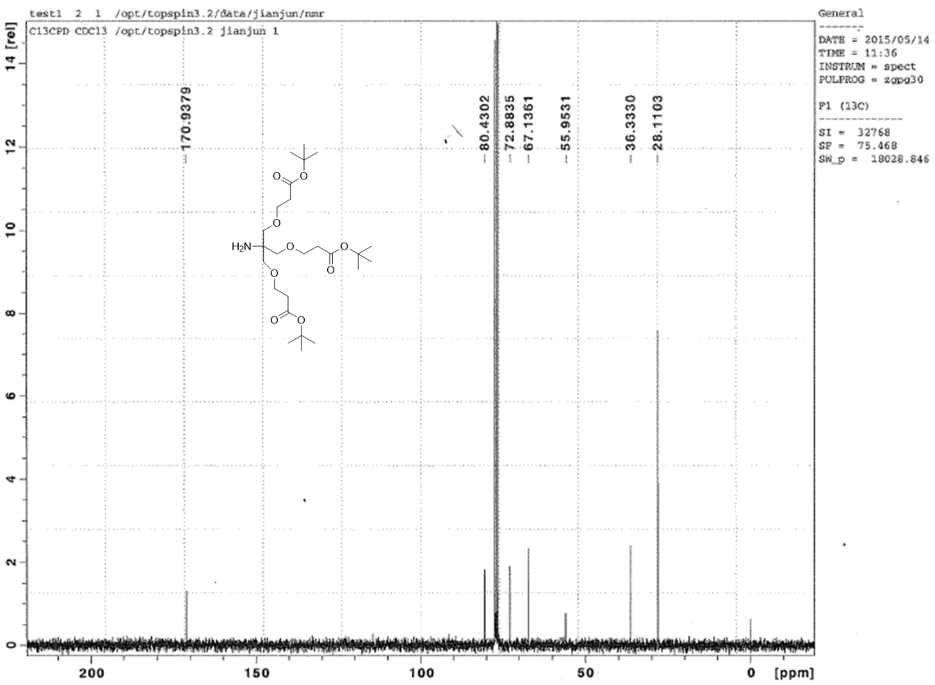

Figure S3. The 13C NMR spectrum of compound 2.

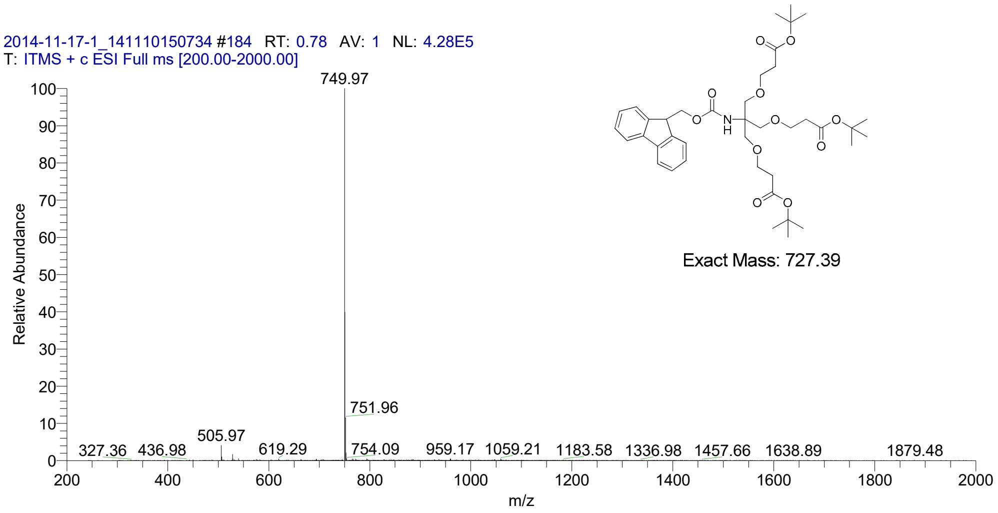

Figure S4. The mass spectrum of compound 3.

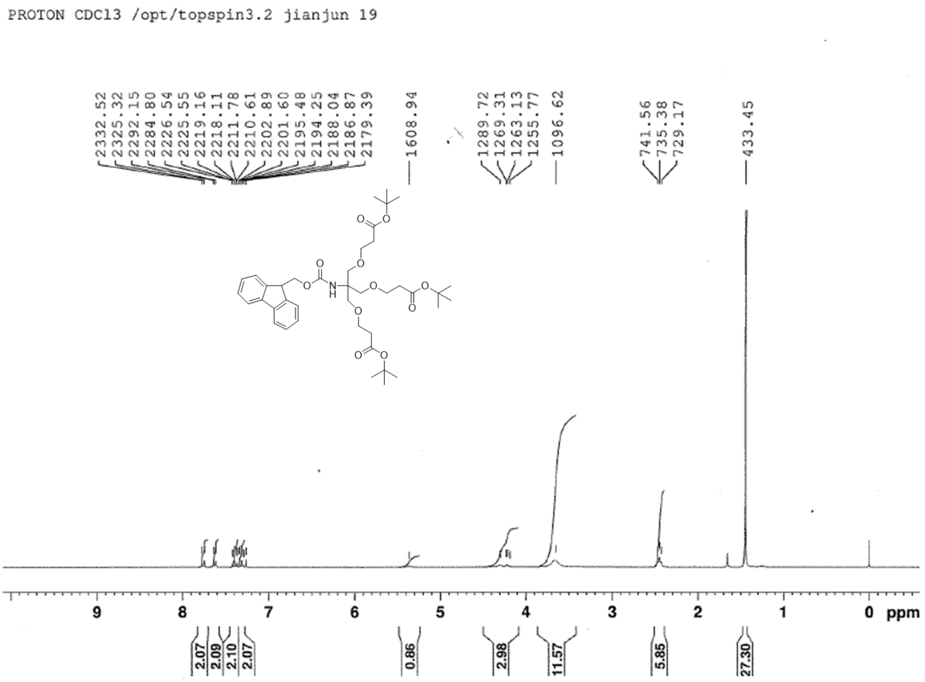

Figure S5. The 1H NMR spectrum of compound 3.

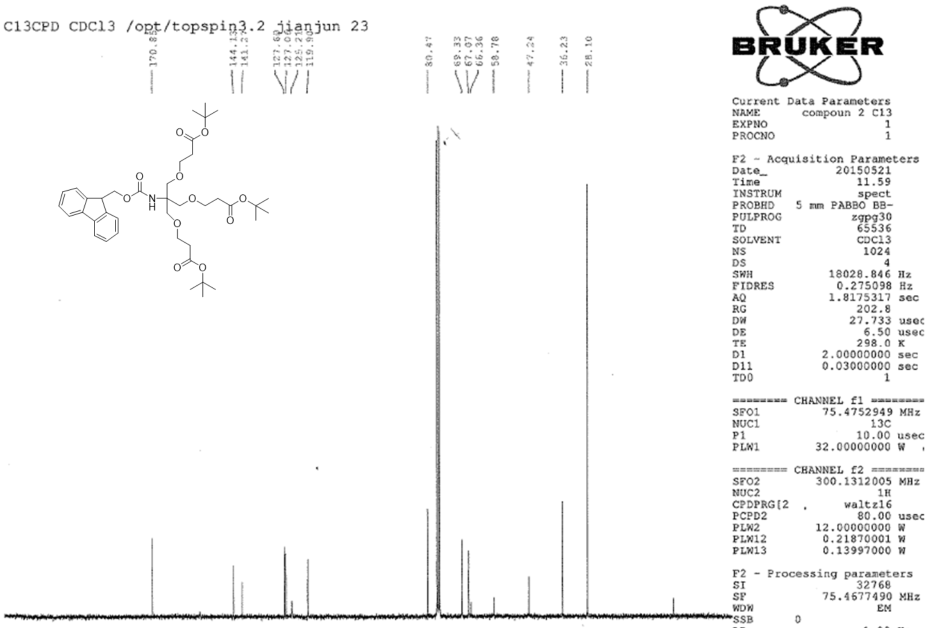

Figure S6. The 13C NMR spectrum of compound 3.

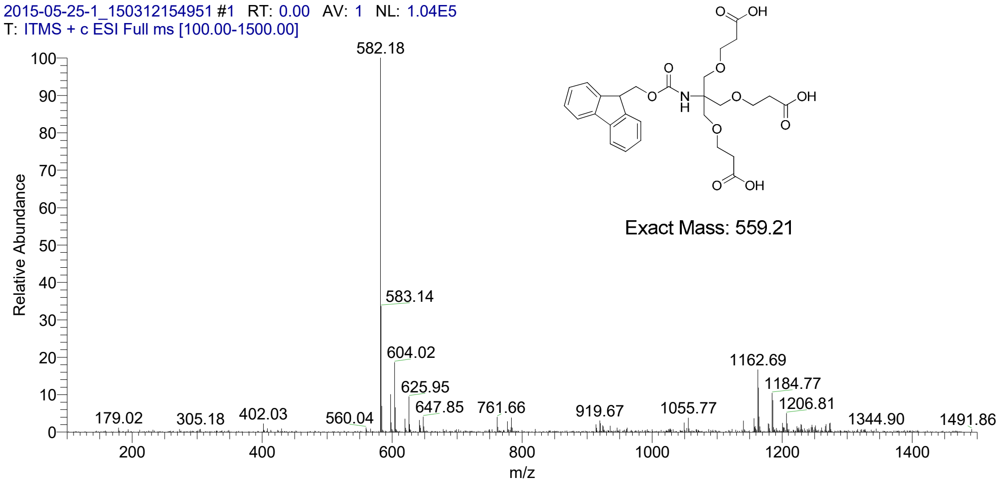

Figure S7. The mass spectrum of compound 4.

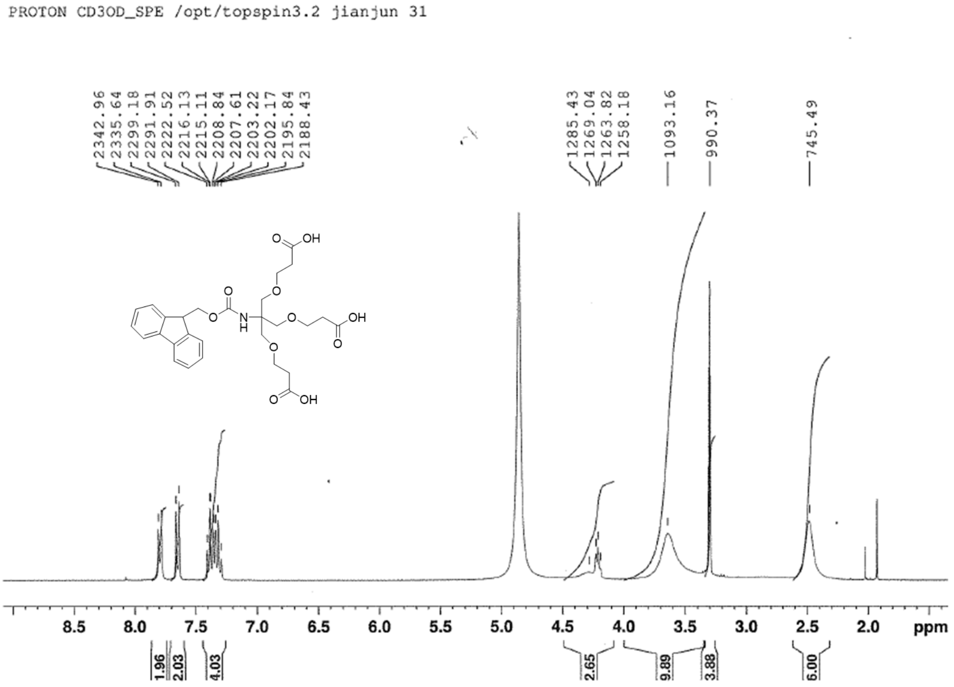

Figure S8. The 1H NMR spectrum of compound 4.

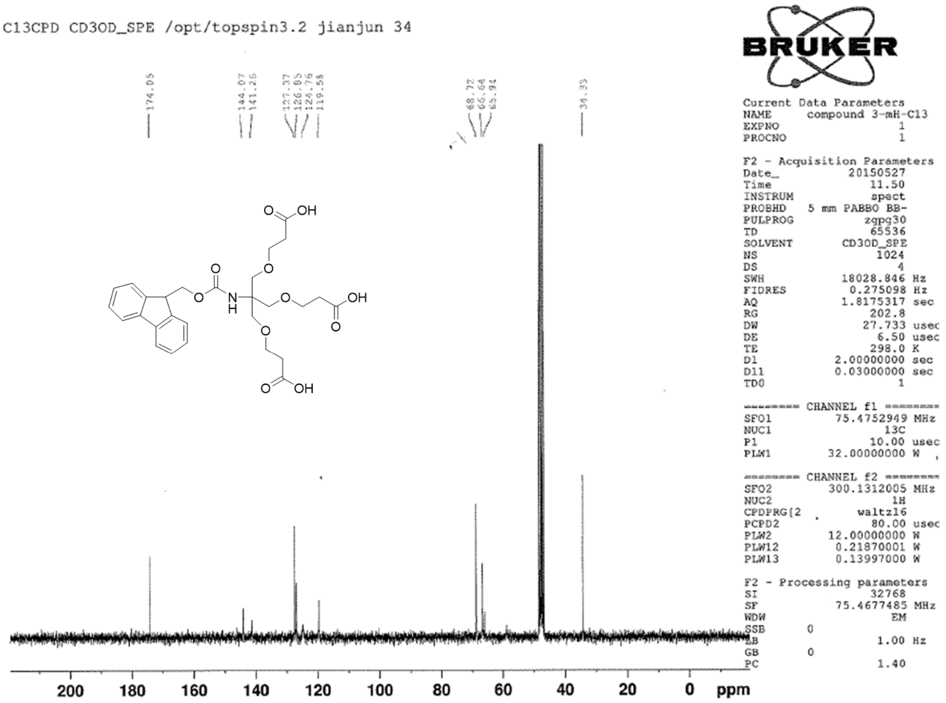

Figure S9. The 13C NMR spectrum of compound 4.

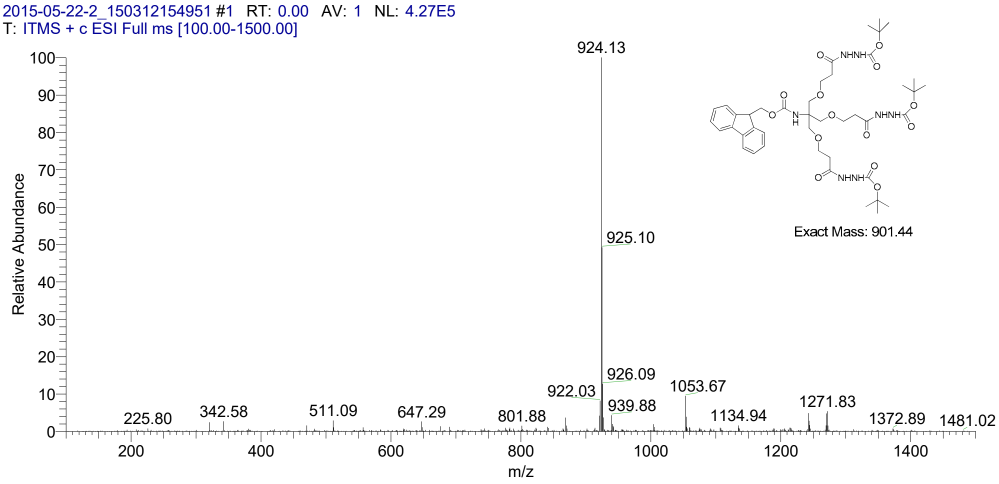

Figure S10. The mass spectrum of compound 5.

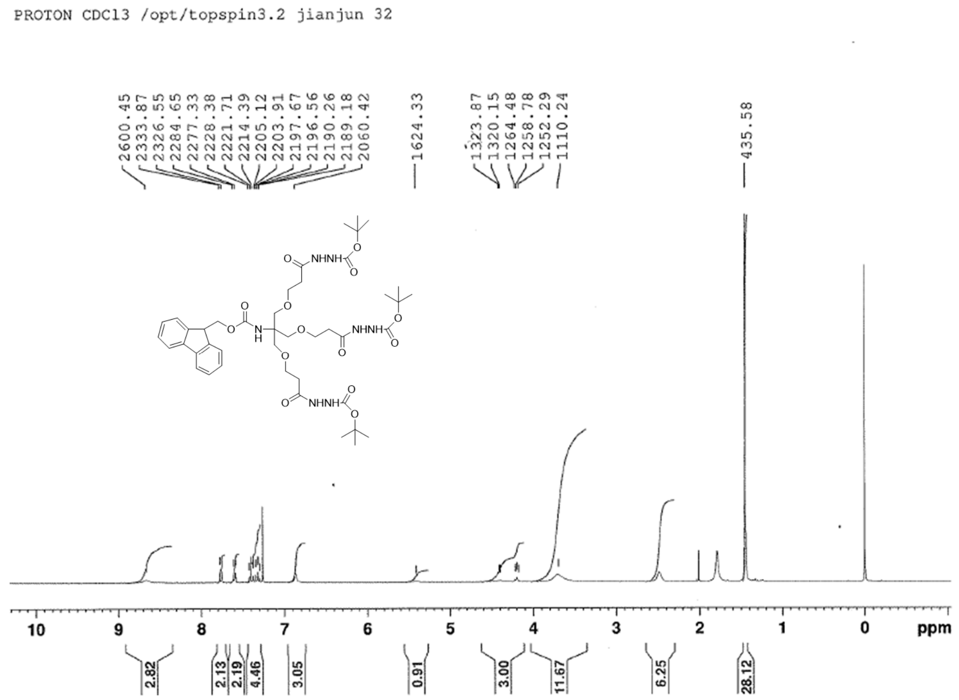

Figure S11. The 1H NMR spectrum of compound 5.

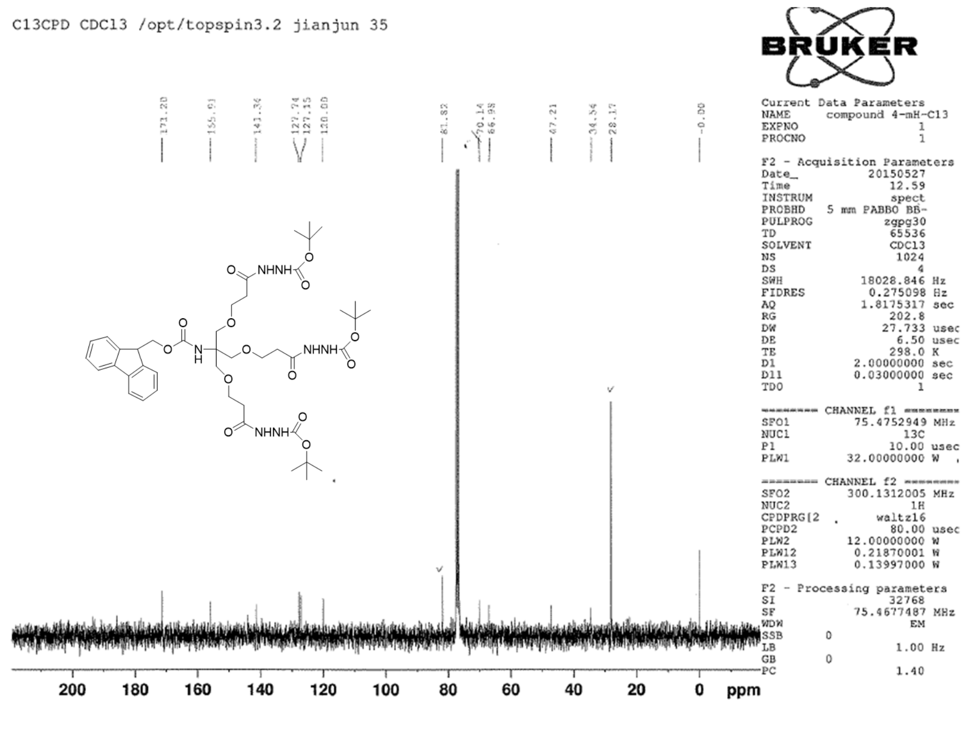

Figure S12. The 13C NMR spectrum of compound 5.

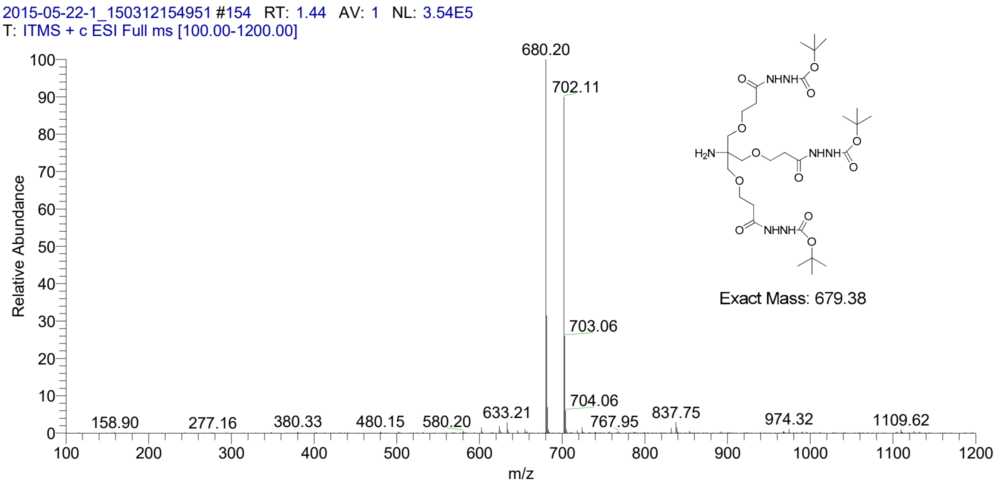

Figure S13. The mass spectrum of compound 6.

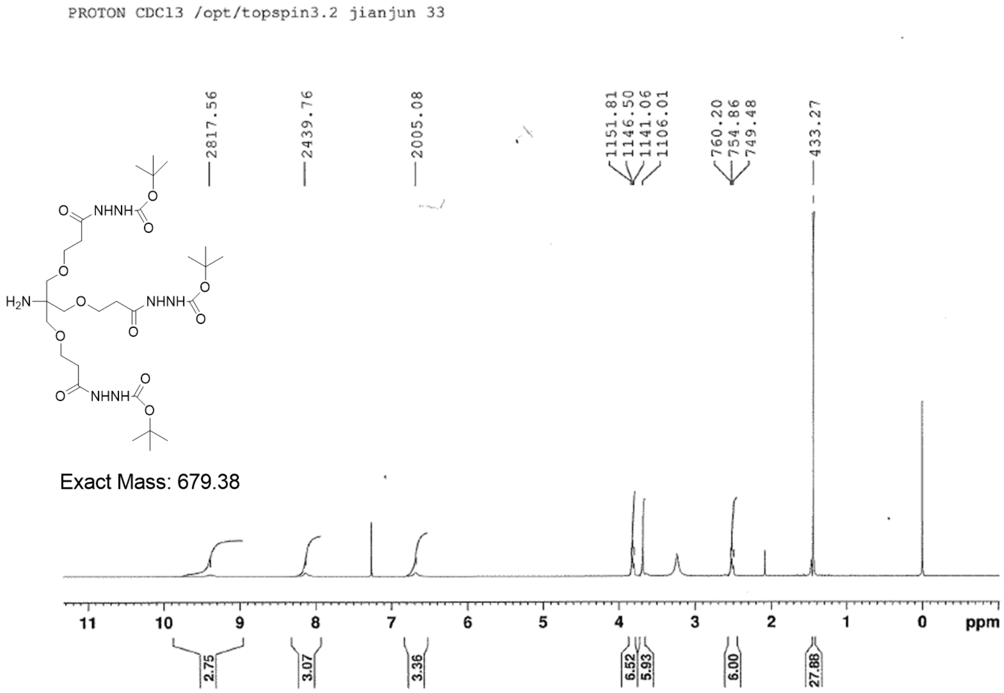

Figure S14. The 1H NMR spectrum of compound 6.

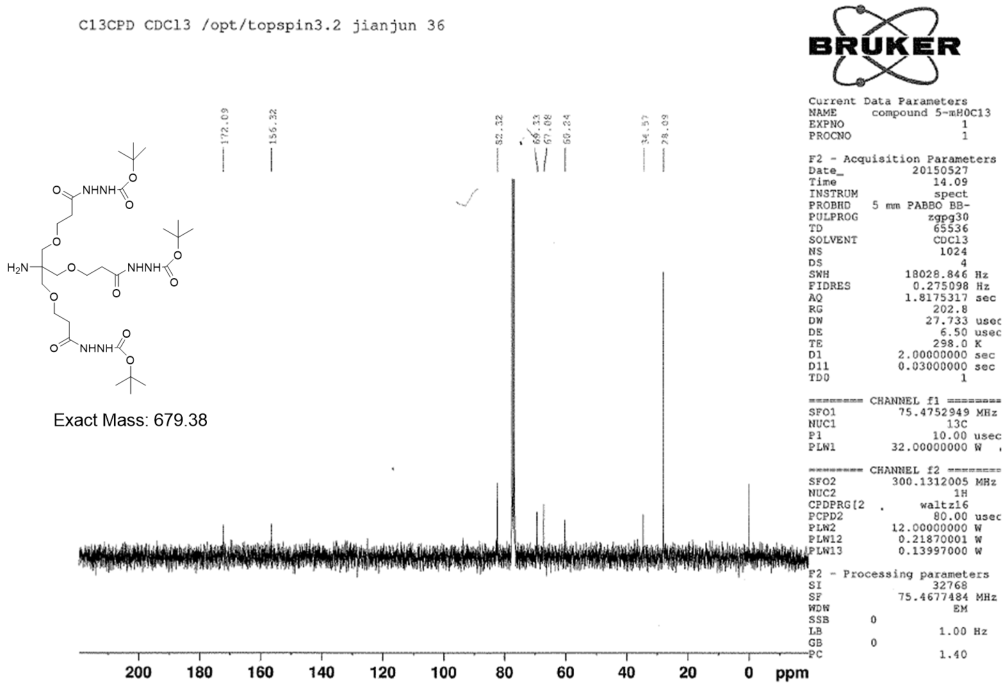

Figure S15. The 13C NMR spectrum of compound 6.

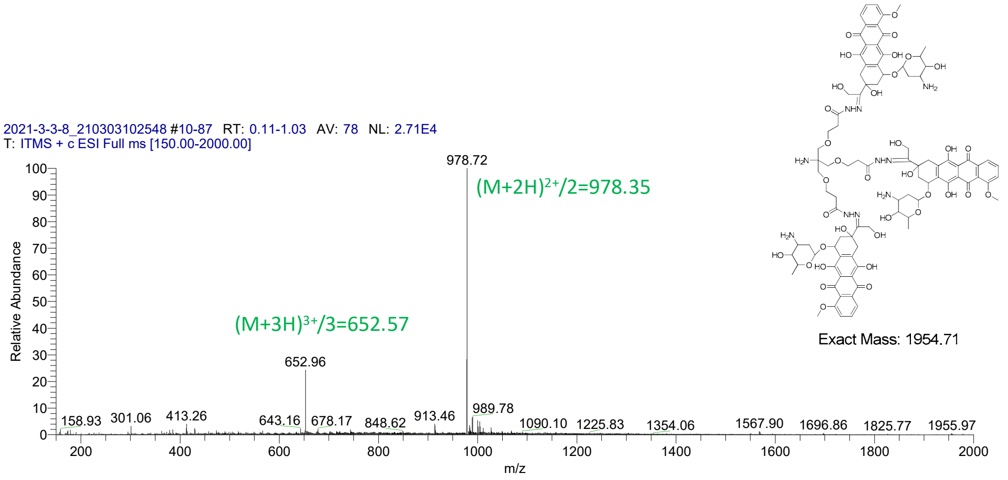

Figure S16. The mass spectrum of TNM-DOX.
